# Supplementary material for: Psychological detachment from work predicts mental wellbeing of working-age adults: Findings from the ‘Wellbeing of the Workforce’ (WoW) prospective longitudinal cohort study
Source: PLoS One. 2025 Jan 14;20(1):e0312673. doi: 10.1371/journal.pone.0312673 (PMC11731735; doi:10.1371/journal.pone.0312673)
Supplement: S1 Text — (PDF) [file pone.0312673.s001.pdf]

The Well-being of the Workforce Study

Participant Information and Consent

Project Title: The Well-being of the Workforce during the COVID-19 Crisis: a mixed method study

Research Team:

• Dr Louise Thomson – louise.thomson@nottingham.ac.uk

• Dr Juliet Hassard – juliet.hassard@nottingham.ac.uk

• Wei Choo – wei.choo@nottingham.ac.uk

• Dr Maria Karanika-Murray – maria.karanika-murray@ntu.ac.uk

• Dr Holly Blake – holly.blake@nottingham.ac.uk

• Rich Pickford – richard.pickford@ntu.ac.uk

Ethics Reference Number: 03-0420

Why have you been invited?

You are being invited to participate in a study that investigates the well-being of the workforce during the COVID-19 pandemic. This is because you are employed, furloughed, self-employed, made redundant due to COVID-19 lockdown, or unemployed prior to the lockdown. The information we get from this study may help us to understand the impact of COVID-19 on the well-being of the workforce, and which factors may minimise any negative effects.

What is the purpose of the study?

The purpose of this research study is to look at how people's work and employment in the UK has changed because of COVID-19, the support they are getting during this period, and how their well-being has been affected.

Your participation in this study is entirely voluntary and you can withdraw at any time by clicking the browser exit button. The data will only be uploaded on completion of the questionnaire by clicking the SUBMIT button. You are free to omit any question.

What you will be asked to do?

The survey will take you approximately 15-20 minutes to complete. We would also like you to answer some shorter follow-up surveys, so we can see how things change over time.

These will be every three months until April 2021.

However, if the situation regarding lockdown and employment restrictions significantly changes we may need to adjust the planned schedule of follow-up surveys.

How will we follow up with you in future?

We will ask you for an email address so that we can contact you for the follow-up surveys. You will also be asked if you are interested in taking part in a further interview. Once again, it is up to you to decide whether or not to take part in the follow-up surveys and interview.

If you do want to take part, we will send you an invitation using the email address that you provide. If you participate in all of the follow-up questionnaires, you will be given an option to be entered into a prize draw to win £250 in high street vouchers.

You can also receive a summary of the findings from this study by visiting the study website at: www.institutemh.org.uk/WoW and signing up to receive the study newsletter.

Are there any risks to taking part?

We believe there are no known risks associated with this research study. However, some of the questions in the survey will ask about your health and well-being, and changes to your work that may be considered sensitive.

If you find any of the questions distressing, we suggest that you contact support services (e.g., Samaritans, 116123; Mind, www.mind.org.uk, 0300 123 3393; ACAS, www.acas.org.uk 0300 123 1100; Anxiety UK, www.anxietyuk.org.uk, 03444775774).

As with any online related activity, the risk of a breach is always possible. We will do everything possible to ensure your answers in this study will remain anonymous. We will

minimise any risks by assigning a randomly generated code to your data so you will not be identifiable during the data analysis process.

All information you provide in the online questionnaire will be handled by the study team and stored by authorised persons from the University of Nottingham who are organising the study. They may also be looked at by authorised people from regulatory organisations to check that the study is being carried out correctly. All will have a duty of confidentiality to you as a study participant and we will do our best to meet this duty.

The results of the study may be published in scientific journals and presented at scientific conferences. The data will be reported anonymously, with any identifying information removed.

Under UK Data Protection laws the University is the Data Controller (legally responsible for the data security) and the Chief Investigator of this study (named above) is the Data Custodian (manages access to the data). This means we are responsible for looking after your information and using it properly. Your rights to access, change or move your information are limited as we need to manage your information in specific ways to comply with certain laws and for the study to be reliable and accurate. To safeguard your rights we will use the minimum personally-identifiable information possible. You can find out more about how we use your information and read our privacy notice at: https://www.nottingham.ac.uk/utilities/privacy.aspx.

At the end of the project, all raw data will be kept securely by the University under the terms of its data protection policy after which it will be disposed of securely. The data will not be kept elsewhere.

What if I have questions about the study?

If you have any questions about this project, please feel free to contact the Lead Researchers: Drs Juliet Hassard (juliet.hassard@nottingham.ac.uk) or Louise Thomson (louise.thomson@nottingham.ac.uk).

If you remain unhappy and wish to complain formally, you should then contact the FMHS Research Ethics Committee Administrator, c/o The University of Nottingham, Faculty PVC Office, B Floor, Medical School, Queen’s Medical Centre Campus, Nottingham University Hospitals, Nottingham, NG7 2UH. E-mail: FMHS-ResearchEthics@nottingham.ac.uk

This study has been reviewed and given a favourable opinion by the University of Nottingham, Faculty of Medicine & Health Sciences Research Ethics Committee [03-0420].

I have read and understood the above information, I confirm that I am 18 years old or older and living and working in the United Kingdom. By clicking the ‘next’ button to begin the online questionnaire, I indicate my willingness to voluntarily take part in the study.

Follow-up contact information

What is the email address you would like us to send the follow-up surveys? This will NOT be shared with third parties. To ensure your anonymity, we will keep this information securely and separately from the information you provide throughout the rest of the survey.

If you found any of the questions distressing, we suggest that you contact support services such as Samaritans (116123), Mind (www.mind.org.uk 0300 123 3393), ACAS (www.acas.org.uk 0300 123 1100), Anxiety UK (www.anxietyuk.org.uk 03444775774).

Demographic Information

Please provide us with some basic information about yourself and those you are living with.

What is your age?

What is your gender?

What is your ethnic group?

If you selected Other ethnic groups, please specify:

What is your geographical location in the United Kingdom?

What is your relationship status?

Are you self-isolating:

If you selected Other, please specify:

Are you self-isolating in a:

If you selected Other, please specify:

How many people are you living with? This is excluding yourself. If none, please indicate with 0.

Are you living with your partner or significant other?

- Yes
- No
- Prefer not to say

Do you have caring responsibilities for dependents?

- Yes
- No
- Prefer not to say

Who do you have caring responsibilities for?

- Child or children under the age of 18
- Elderly parent(s)
- Both
- Other

If you selected Other, please specify:

How many dependents do you have living with you?

What are the ages of the dependents living with you? (if more than one, separate their ages with a comma, e.g. 2, 8, 12, 72, 88)

Which of the following most accurately describes your current work status for your main job? The answer to this question is required. This is to allow us to tailor the survey to your situation.

- Employed and working
- Employed and furloughed
- Made redundant due to COVID-19 lockdown
- Self-employed and still working
- Self-employed and not working
- Unemployed prior to COVID-19 lockdown
- On a zero-hours contract
- Other

If you selected Other, please specify:

If you found any of the questions distressing, we suggest that you contact support services such as Samaritans (116123), Mind (www.mind.org.uk 0300 123 3393), ACAS (www.acas.org.uk 0300 123 1100), Anxiety UK (www.anxietyuk.org.uk 03444775774).

Your work and employment contract

In this section, we will ask you a series of questions about your employment contract and the nature of your work.

What is the nature of your employment contract?

If you selected Other, please specify:

How many hours are you employed to work a week as stated on your contract?

What were your average weekly hours before lockdown?

What are your average weekly hours during lockdown?

Please select the option that best represents what you do in your main paid job? By main job, we mean the one you spend most of your working hours.

If you selected Other, please specify:

Please select the option that best describes the industry your organisation is in.

If you selected Other, please specify:

Are you working in the…?

If you selected Other, please specify:

How would you describe the size of your organisation?

Are you a key worker?

Do you manage or supervise anyone at work?

How many people work under your supervision?

How many years have you worked in this organisation? If less than a year, please indicate with 0.

If you found any of the questions distressing, we suggest that you contact support services such as Samaritans (116123), Mind (www.mind.org.uk 0300 123 3393), ACAS (www.acas.org.uk 0300 123 1100), Anxiety UK (www.anxietyuk.org.uk 03444775774).

Your work and employment contract

In this section, we will ask you a series of questions about your employment contract and the nature of your work.

What is the nature of your employment contract?

If you selected Other, please specify:

How many hours are you employed to work a week as stated on your contract?

Please select the option that best represents what you do in your main paid job? By main job, we mean the one you spend most of your working hours.

If you selected Other, please specify:

Please select the option that best describes the industry your organisation is in.

If you selected Other, please specify:

Are you working in the…?

If you selected Other, please specify:

How would you describe the size of your organisation?

Are you a key worker?

Do you manage or supervise anyone at work?

How many people work under your supervision?

How many years have you worked in this organisation? If less than a year, please indicate with 0.

If you found any of the questions distressing, we suggest that you contact support services such as Samaritans (116123), Mind (www.mind.org.uk 0300 123 3393), ACAS (www.acas.org.uk 0300 123 1100), Anxiety UK (www.anxietyuk.org.uk 03444775774).

Your work and employment contract

In this section, we will ask you about the nature of your self-employment.

Which of the following describes the nature of your self-employment? You may select more than one option.

- ☐ Sole trader
- ☐ Business owner or partner with employees
- ☐ Doing freelance work
- ☐ Sub-contractor or contract worker
- ☐ Work through an agency
- ☐ Employed on a zero-hours contract
- ☐ Other

If you selected Other, please specify:

____

If you found any of the questions distressing, we suggest that you contact support services such as Samaritans (116123), Mind (www.mind.org.uk 0300 123 3393), ACAS (www.acas.org.uk 0300 123 1100), Anxiety UK (www.anxietyuk.org.uk 03444775774).

Your work and employment contract

In this section, we will ask you about the nature of your self-employment.

Which of the following describes the nature of your self-employment? You may select more than one option.

- ☐ Sole trader
- ☐ Business owner or partner with employees
- ☐ Doing freelance work
- ☐ Sub-contractor or contract worker
- ☐ Work through an agency
- ☐ Employed on a zero-hours contract
- ☐ Other

If you selected Other, please specify:

____

If you found any of the questions distressing, we suggest that you contact support services such as Samaritans (116123), Mind (www.mind.org.uk 0300 123 3393), ACAS (www.acas.org.uk 0300 123 1100), Anxiety UK (www.anxietyuk.org.uk 03444775774).

Your well-being

In this section, we will ask you a series of questions about your overall well-being.

Please indicate for each of the five statements, which is closest to how you have been feeling over the last two weeks.

	All of the time	Most of the time	More than half of the time	Less than half of the time	Some of the time	At no time
I have felt cheerful and in good spirits.	☐	☐	☐	☐	☐	☐
I have felt calm and relaxed.	☐	☐	☐	☐	☐	☐
I have felt active and vigorous.	☐	☐	☐	☐	☐	☐
I woke up feeling fresh and rested.	☐	☐	☐	☐	☐	☐
My daily life has been filled with things that interest me.	☐	☐	☐	☐	☐	☐

In general, how satisfied are you with your life?

How is your health in general? Would you say it is…

Over the last 2 weeks, how often have you been bothered by the following problems?

	Not at all	Several days	More than half the days	Nearly every day
Feeling nervous, anxious or on edge	☐	☐	☐	☐
Not being able to stop or control worrying	☐	☐	☐	☐
Worrying too much about different things	☐	☐	☐	☐
Trouble relaxing	☐	☐	☐	☐
Being so restless that it is hard to sit still	☐	☐	☐	☐
Becoming easily annoyed or irritable	☐	☐	☐	☐
Feeling afraid as if something awful might happen	☐	☐	☐	☐

Since the start of lockdown, please indicate to what degree you agree with the following statements:

	I strongly agree	I agree	Mixed or neither agree nor disagree	I disagree	I strongly disagree
I forget about work.	☐	☐	☐	☐	☐
I don't think about work at all.	☐	☐	☐	☐	☐
I distance myself from my work.	☐	☐	☐	☐	☐
I get a break from the demands of work.	☐	☐	☐	☐	☐

If you found any of the questions distressing, we suggest that you contact support services such as Samaritans (116123), Mind (www.mind.org.uk 0300 123 3393), ACAS (www.acas.org.uk 0300 123 1100), Anxiety UK (www.anxietyuk.org.uk 03444775774).

Your well-being

In this section, we will ask you a series of questions about your overall well-being.

Please indicate for each of the five statements, which is closest to how you have been feeling over the last two weeks.

	All of the time	Most of the time	More than half of the time	Less than half of the time	Some of the time	At no time
I have felt cheerful and in good spirits.	☐	☐	☐	☐	☐	☐
I have felt calm and relaxed.	☐	☐	☐	☐	☐	☐
I have felt active and vigorous.	☐	☐	☐	☐	☐	☐
I woke up feeling fresh and rested.	☐	☐	☐	☐	☐	☐
My daily life has been filled with things that interest me.	☐	☐	☐	☐	☐	☐

In general, how satisfied are you with your life?

How is your health in general? Would you say it is…

Over the last 2 weeks, how often have you been bothered by the following problems?

	Not at all	Several days	More than half the days	Nearly every day
Feeling nervous, anxious or on edge	☐	☐	☐	☐
Not being able to stop or control worrying	☐	☐	☐	☐
Worrying too much about different things	☐	☐	☐	☐
Trouble relaxing	☐	☐	☐	☐
Being so restless that it is hard to sit still	☐	☐	☐	☐
Becoming easily annoyed or irritable	☐	☐	☐	☐
Feeling afraid as if something awful might happen	☐	☐	☐	☐

If you found any of the questions distressing, we suggest that you contact support services such as Samaritans (116123), Mind (www.mind.org.uk 0300 123 3393), ACAS (www.acas.org.uk 0300 123 1100), Anxiety UK (www.anxietyuk.org.uk 03444775774).

Your well-being

In this section, we will ask you a series of questions about your overall well-being.

Please indicate for each of the five statements, which is closest to how you have been feeling over the last two weeks.

	All of the time	Most of the time	More than half of the time	Less than half of the time	Some of the time	At no time
I have felt cheerful and in good spirits.	☐	☐	☐	☐	☐	☐
I have felt calm and relaxed.	☐	☐	☐	☐	☐	☐
I have felt active and vigorous.	☐	☐	☐	☐	☐	☐
I woke up feeling fresh and rested.	☐	☐	☐	☐	☐	☐
My daily life has been filled with things that interest me.	☐	☐	☐	☐	☐	☐

In general, how satisfied are you with your life?

How is your health in general? Would you say it is…

Over the last 2 weeks, how often have you been bothered by the following problems?

	Not at all	Several days	More than half the days	Nearly every day
Feeling nervous, anxious or on edge	☐	☐	☐	☐
Not being able to stop or control worrying	☐	☐	☐	☐
Worrying too much about different things	☐	☐	☐	☐
Trouble relaxing	☐	☐	☐	☐
Being so restless that it is hard to sit still	☐	☐	☐	☐
Becoming easily annoyed or irritable	☐	☐	☐	☐
Feeling afraid as if something awful might happen	☐	☐	☐	☐

Since the start of lockdown, please indicate to what degree you agree with the following statements:

	I strongly agree	I agree	Mixed or neither agree nor disagree	I disagree	I strongly disagree
I forget about work.	☐	☐	☐	☐	☐
I don't think about work at all.	☐	☐	☐	☐	☐
I distance myself from my work.	☐	☐	☐	☐	☐
I get a break from the demands of work.	☐	☐	☐	☐	☐

If you found any of the questions distressing, we suggest that you contact support services such as Samaritans (116123), Mind (www.mind.org.uk 0300 123 3393), ACAS (www.acas.org.uk 0300 123 1100), Anxiety UK (www.anxietyuk.org.uk 03444775774).

Your well-being

In this section, we will ask you a series of questions about your overall well-being.

Please indicate for each of the five statements, which is closest to how you have been feeling over the last two weeks.

	All of the time	Most of the time	More than half of the time	Less than half of the time	Some of the time	At no time
I have felt cheerful and in good spirits.	☐	☐	☐	☐	☐	☐
I have felt calm and relaxed.	☐	☐	☐	☐	☐	☐
I have felt active and vigorous.	☐	☐	☐	☐	☐	☐
I woke up feeling fresh and rested.	☐	☐	☐	☐	☐	☐
My daily life has been filled with things that interest me.	☐	☐	☐	☐	☐	☐

In general, how satisfied are you with your life?

How is your health in general? Would you say it is…

Over the last 2 weeks, how often have you been bothered by the following problems?

	Not at all	Several days	More than half the days	Nearly every day
Feeling nervous, anxious or on edge	☐	☐	☐	☐
Not being able to stop or control worrying	☐	☐	☐	☐
Worrying too much about different things	☐	☐	☐	☐
Trouble relaxing	☐	☐	☐	☐
Being so restless that it is hard to sit still	☐	☐	☐	☐
Becoming easily annoyed or irritable	☐	☐	☐	☐
Feeling afraid as if something awful might happen	☐	☐	☐	☐

If you found any of the questions distressing, we suggest that you contact support services such as Samaritans (116123), Mind (www.mind.org.uk 0300 123 3393), ACAS (www.acas.org.uk 0300 123 1100), Anxiety UK (www.anxietyuk.org.uk 03444775774).

Your work, working conditions and organisation

This series of questions will ask you about your work or working conditions. Please select the response that best represents your experience.

Please select the response that best represents your experience at work. My work has changed in the following ways since the start of lockdown.

	Increased a lot	Increased slightly	Same as usual	Decreased slightly	Decreased a lot
The number of hours you work per week.	☐	☐	☐	☐	☐
Your salary or income.	☐	☐	☐	☐	☐
The amount of influence you have over your work.	☐	☐	☐	☐	☐
Your task and duties.	☐	☐	☐	☐	☐

Please select the response that best represents your experiences and feelings at work at the moment.

	Always	Often	Sometimes	Seldom	Never/ hardly ever
Is your workload unevenly distributed so it piles up?	☐	☐	☐	☐	☐
How often do you not have time to complete all your work tasks?	☐	☐	☐	☐	☐
Do you get behind with your work?	☐	☐	☐	☐	☐

Do you have enough time for your work tasks?	☐	☐	☐	☐	☐

Please select the response that best represents your experiences and feelings at work at the moment.

	Always	Often	Sometimes	Seldom	Never/ hardly ever
Do you have a large degree of influence on the decisions concerning your work?	☐	☐	☐	☐	☐
Can you influence the amount of work assigned to you?	☐	☐	☐	☐	☐
Do you have any influence on what you do at work?	☐	☐	☐	☐	☐
Do you have any influence on how you do your work?	☐	☐	☐	☐	☐

Please select the response that best represents your experiences and feelings at work at the moment.

	Always	Often	Sometimes	Seldom	Never/ hardly ever
Are you worried about becoming unemployed?	☐	☐	☐	☐	☐
Are you worried about being made redundant?	☐	☐	☐	☐	☐

Are you worried about it being difficult for you to find another job if you became unemployed?	☐	☐	☐	☐	☐

Please indicate the option that best corresponds with your personal experience at the moment.

	Always	Often	Sometimes	Seldom	Never/hardly ever
My organisation has many programs and policies designed to help employees balance work and family life.	☐	☐	☐	☐	☐
My organisation makes an active effort to help employees when there is a conflict between work and family life.	☐	☐	☐	☐	☐
My organisation puts money and effort into showing its support of employees and families.	☐	☐	☐	☐	☐
It is easy to find out about family support programs within my organisation.	☐	☐	☐	☐	☐

My organisation provides its employees with useful information they need to balance work and family.	☐	☐	☐	☐	☐
My organisation helps employees with families to find the information they need to balance work and family.	☐	☐	☐	☐	☐
My organisation is understanding when an employee has a conflict between work and family.	☐	☐	☐	☐	☐
In general, my organisation is very supportive of its employees with families.	☐	☐	☐	☐	☐
Employees really feel that the organisation respects their desire to balance work and family demands.	☐	☐	☐	☐	☐
My organisation is more family-friendly than most other organisations I could work for.	☐	☐	☐	☐	☐

Please indicate the option that best corresponds with your feelings about work at the moment.

	To a very large extent	To a large extent	Somewhat	To a small extent	To a very small extent
Do you enjoy telling others about your place of work?	☐	☐	☐	☐	☐
Do you feel that your place of work is of great importance to you?	☐	☐	☐	☐	☐
Would you recommend other people to apply for a position at your workplace?	☐	☐	☐	☐	☐

These following questions aim to examine your feelings about your organisation. Please read each question and select the response that best represents your personal experience.

How often have you considered leaving your job?

To what extent is your current job satisfying your personal needs?

How often are you frustrated when not given the opportunity at work to achieve your personal work-related goals?

How often do you dream about getting another job that will better suit your personal needs?

How likely are you to accept another job at the same level of pay should it be offered to you?

How often do you look forward to another day at work?

The next five questions concern the ways in which your work affects your private life. Please select the response that best represents your experience at the moment.

	To a very large extent	To a large extent	To a moderate extent	To a small extent	To a very small extent
Are there times when you need to be attending to work tasks and home tasks at the same time?	☐	☐	☐	☐	☐

Do you feel that your work drains so much of your energy that it has a negative effect on your private life?	☐	☐	☐	☐	☐
Do you feel that your work takes so much of your time that it has a negative effect on your private life?	☐	☐	☐	☐	☐
The demands of my work interfere with my private and family life.	☐	☐	☐	☐	☐
Due to work-related duties, I have to make changes to my plans for private and family activities.	☐	☐	☐	☐	☐

If you found any of the questions distressing, we suggest that you contact support services such as Samaritans (116123), Mind (www.mind.org.uk 0300 123 3393), ACAS (www.acas.org.uk 0300 123 1100), Anxiety UK (www.anxietyuk.org.uk 03444775774).

Your work, working conditions and organisation

This series of questions will ask you about your work or working conditions. Please select the response that best represents your experience.

Please select the response that best represents your experience during the furloughing process.

	Strongly agree	Agree	Mixed or neither agree nor disagree	Disagree	Strongly disagree
Do you feel that you completely understand the reasons that brought about being furloughed?	☐	☐	☐	☐	☐
Do you feel that your organisation provides you with the necessary information to understand the reasons behind being furloughed?	☐	☐	☐	☐	☐
Do you understand why the furlough process was implemented in the way that it was?	☐	☐	☐	☐	☐

Do you feel that your organisation took into account your opinions and ideas in the implementation of the furlough process?	☐	☐	☐	☐	☐
Do you feel that your organisation cares about your worries towards being furloughed?	☐	☐	☐	☐	☐
To what extent were your worries taken into account before you were furloughed?	☐	☐	☐	☐	☐
Do you feel that you personally have control over the furlough process?	☐	☐	☐	☐	☐
Do you have the opportunity to propose ways of influencing the furlough process?	☐	☐	☐	☐	☐
Do you feel that you personally have influence in the way the furlough process was implemented?	☐	☐	☐	☐	☐

Please select the response that best represents your experiences and feelings at work at the moment.

	Always	Often	Sometimes	Seldom	Never/ hardly ever
Are you worried about becoming unemployed?	☐	☐	☐	☐	☐
Are you worried about being made redundant?	☐	☐	☐	☐	☐
Are you worried about it being difficult for you to find another job if you became unemployed?	☐	☐	☐	☐	☐

Please indicate the option that best corresponds with your personal experience at the moment.

	Always	Often	Sometimes	Seldom	Never/hardly ever
My organisation has many programs and policies designed to help employees balance work and family life.	☐	☐	☐	☐	☐
My organisation makes an active effort to help employees when there is a conflict between work and family life.	☐	☐	☐	☐	☐

My organisation puts money and effort into showing its support of employees and families.	☐	☐	☐	☐	☐
It is easy to find out about family support programs within my organisation.	☐	☐	☐	☐	☐
My organisation provides its employees with useful information they need to balance work and family.	☐	☐	☐	☐	☐
My organisation helps employees with families to find the information they need to balance work and family.	☐	☐	☐	☐	☐
My organisation is understanding when an employee has a conflict between work and family.	☐	☐	☐	☐	☐
In general, my organisation is very supportive of its employees with families.	☐	☐	☐	☐	☐

Employees really feel that the organisation respects their desire to balance work and family demands.	☐	☐	☐	☐	☐
My organisation is more family-friendly than most other organisations I could work for.	☐	☐	☐	☐	☐

Please indicate the option that best corresponds with your feelings about work at the moment.

	To a very large extent	To a large extent	Somewhat	To a small extent	To a very small extent
Do you enjoy telling others about your place of work?	☐	☐	☐	☐	☐
Do you feel that your place of work is of great importance to you?	☐	☐	☐	☐	☐
Would you recommend other people to apply for a position at your workplace?	☐	☐	☐	☐	☐

These following questions aim to examine your feelings about your organisation. Please read each question and select the response that best represents your personal experience.

How often have you considered leaving your job?

To what extent is your current job satisfying your personal needs?

How often are you frustrated when not given the opportunity at work to achieve your personal work-related goals?

How often do you dream about getting another job that will better suit your personal needs?

How likely are you to accept another job at the same level of pay should it be offered to you?

How often do you look forward to another day at work?

If you found any of the questions distressing, we suggest that you contact support services such as Samaritans (116123), Mind

(www.mind.org.uk 0300 123 3393), ACAS (www.acas.org.uk 0300 123 1100), Anxiety UK (www.anxietyuk.org.uk 03444775774).

Your work, working conditions and organisation

This series of questions will ask you about your work or working conditions. Please select the response that best represents your experience.

Please select the response that best represents your experience at work. My work has changed in the following ways since the start of lockdown.

	Increased a lot	Increased slightly	Same as usual	Decreased slightly	Decreased a lot
The number of hours you work per week.	☐	☐	☐	☐	☐
Your salary or income.	☐	☐	☐	☐	☐
The amount of influence you have over your work.	☐	☐	☐	☐	☐
Your task and duties.	☐	☐	☐	☐	☐

The next five questions concern the ways in which your work affects your private life. Please select the response that best represents your experience at the moment.

	To a very large extent	To a large extent	To a moderate extent	To a small extent	To a very small extent
Are there times when you need to be attending to work tasks and home tasks at the same time?	☐	☐	☐	☐	☐

Do you feel that your work drains so much of your energy that it has a negative effect on your private life?					
Do you feel that your work takes so much of your time that it has a negative effect on your private life?					
The demands of my work interfere with my private and family life.					
Due to work-related duties, I have to make changes to my plans for private and family activities.					

If you found any of the questions distressing, we suggest that you contact support services such as Samaritans (116123), Mind (www.mind.org.uk 0300 123 3393), ACAS (www.acas.org.uk 0300 123 1100), Anxiety UK (www.anxietyuk.org.uk 03444775774).

Experiences and supports at home

This section will ask you a series of questions about your experiences at home and forms of social support important to you.

Please select the response that best represents your personal experience at the moment.

	Always	Very Often	Sometimes	Rarely	Never
Do you find that you are busy at home?	☐	☐	☐	☐	☐
Do you have to do many things in a hurry when you are at home?	☐	☐	☐	☐	☐
Do you have to carry out a lot of tasks at home (household/caring tasks)?	☐	☐	☐	☐	☐
How often do issues arise at home that are emotionally demanding?	☐	☐	☐	☐	☐
How often does your housework confront you with things that touch you personally?	☐	☐	☐	☐	☐
How often do you get frustrated about things concerning your home-life?	☐	☐	☐	☐	☐

Do you find that you have to plan and organise a lot of things in relation to your home life?	☐	☐	☐	☐	☐
Do you have to remember a lot of things with regard to your home life?	☐	☐	☐	☐	☐
Do you have to do many things simultaneously at home?	☐	☐	☐	☐	☐
Do you have to coordinate everything carefully at home?	☐	☐	☐	☐	☐

How financially secure do you feel at the moment?

Think about a person whom you are closest to during lockdown, who is either your partner or a family member.

	Very often	Often	Sometimes	Seldom	Never
How often did he/she make you feel good about yourself?	☐	☐	☐	☐	☐
How often do you share hobbies or other pleasant things together?	☐	☐	☐	☐	☐

How often did he/she give you stress or worries?	☐	☐	☐	☐	☐
How often do you trust him/her with your most private problems?	☐	☐	☐	☐	☐
How often would you have liked to confide in him/her more?	☐	☐	☐	☐	☐
How often did you feel bad after talking with him/her?	☐	☐	☐	☐	☐
How often did he/she share personal problems with you?	☐	☐	☐	☐	☐
How often did you need his/her practical assistance with important matters?	☐	☐	☐	☐	☐
How often did you actually receive this practical assistance with important matters from him/her?	☐	☐	☐	☐	☐
How often did you want more practical assistance from him/her?	☐	☐	☐	☐	☐

Is this partner or family member living with you?

- ○ Yes
- ○ No
- ○ Prefer not to say

Think about a person who you are closest to during lockdown, who is neither your partner nor a family member. This could be a friend or work colleague, for example.

	Very often	Often	Sometimes	Seldom	Never
How often did he/she make you feel good about yourself?	☐	☐	☐	☐	☐
How often do you share hobbies or other pleasant things together?	☐	☐	☐	☐	☐
How often did he/she give you stress or worries?	☐	☐	☐	☐	☐
How often do you trust him/her with your most private problems?	☐	☐	☐	☐	☐
How often would you have liked to confide in him/her more?	☐	☐	☐	☐	☐
How often did you feel bad after talking with him/her?	☐	☐	☐	☐	☐
How often did he/she share personal problems with you?	☐	☐	☐	☐	☐
How often did you need his/her practical assistance with important matters?	☐	☐	☐	☐	☐

How often did you actually receive this practical assistance with important matters from him/her?	☐	☐	☐	☐	☐
How often did you want more practical assistance from him/her?	☐	☐	☐	☐	☐

Is this person who you are closest to (but is not your partner nor a family member) living with you?

- ○ Yes
- ○ No
- ○ Prefer not to say

If you found any of the questions distressing, we suggest that you contact support services such as Samaritans (116123), Mind (www.mind.org.uk 0300 123 3393), ACAS (www.acas.org.uk 0300 123 1100), Anxiety UK (www.anxietyuk.org.uk 03444775774).

Experiences and supports at home

This section will ask you a series of questions about your experiences at home and forms of social support important to you.

Please select the response that best represents your personal experience at the moment.

	Always	Very Often	Sometimes	Rarely	Never
Do you find that you are busy at home?	☐	☐	☐	☐	☐
Do you have to do many things in a hurry when you are at home?	☐	☐	☐	☐	☐
Do you have to carry out a lot of tasks at home (household/caring tasks)?	☐	☐	☐	☐	☐
How often do issues arise at home that are emotionally demanding?	☐	☐	☐	☐	☐
How often does your housework confront you with things that touch you personally?	☐	☐	☐	☐	☐
How often do you get frustrated about things concerning your home-life?	☐	☐	☐	☐	☐

Do you find that you have to plan and organise a lot of things in relation to your home life?	☐	☐	☐	☐	☐
Do you have to remember a lot of things with regard to your home life?	☐	☐	☐	☐	☐
Do you have to do many things simultaneously at home?	☐	☐	☐	☐	☐
Do you have to coordinate everything carefully at home?	☐	☐	☐	☐	☐

How financially secure do you feel at the moment?

Think about a person whom you are closest to during lockdown, who is either your partner or a family member.

	Very often	Often	Sometimes	Seldom	Never
How often did he/she make you feel good about yourself?	☐	☐	☐	☐	☐
How often do you share hobbies or other pleasant things together?	☐	☐	☐	☐	☐

How often did he/she give you stress or worries?	☐	☐	☐	☐	☐
How often do you trust him/her with your most private problems?	☐	☐	☐	☐	☐
How often would you have liked to confide in him/her more?	☐	☐	☐	☐	☐
How often did you feel bad after talking with him/her?	☐	☐	☐	☐	☐
How often did he/she share personal problems with you?	☐	☐	☐	☐	☐
How often did you need his/her practical assistance with important matters?	☐	☐	☐	☐	☐
How often did you actually receive this practical assistance with important matters from him/her?	☐	☐	☐	☐	☐
How often did you want more practical assistance from him/her?	☐	☐	☐	☐	☐

Is this partner or family member living with you?

- ○ Yes
- ○ No
- ○ Prefer not to say

Think about a person who you are closest to during lockdown, who is neither your partner nor a family member. This could be a friend or work colleague, for example.

	Very often	Often	Sometimes	Seldom	Never
How often did he/she make you feel good about yourself?	☐	☐	☐	☐	☐
How often do you share hobbies or other pleasant things together?	☐	☐	☐	☐	☐
How often did he/she give you stress or worries?	☐	☐	☐	☐	☐
How often do you trust him/her with your most private problems?	☐	☐	☐	☐	☐
How often would you have liked to confide in him/her more?	☐	☐	☐	☐	☐
How often did you feel bad after talking with him/her?	☐	☐	☐	☐	☐
How often did he/she share personal problems with you?	☐	☐	☐	☐	☐
How often did you need his/her practical assistance with important matters?	☐	☐	☐	☐	☐

How often did you actually receive this practical assistance with important matters from him/her?	☐	☐	☐	☐	☐
How often did you want more practical assistance from him/her?	☐	☐	☐	☐	☐

Is this person who you are closest to (but is not your partner nor a family member) living with you?

- Yes
- No
- Prefer not to say

If you found any of the questions distressing, we suggest that you contact support services such as Samaritans (116123), Mind (www.mind.org.uk 0300 123 3393), ACAS (www.acas.org.uk 0300 123 1100), Anxiety UK (www.anxietyuk.org.uk 03444775774).

About you

This section will ask you a series of questions about your feelings about life and personal activities.

To what degree have you experienced changes in your feelings as a result of COVID-19?

	A great degree	A moderate degree	Some degree	A small degree	Not at all
A feeling that my future success depends on me.	☐	☐	☐	☐	☐
A sense of optimism.	☐	☐	☐	☐	☐
A feeling that I have control over my life.	☐	☐	☐	☐	☐
A feeling that my life has meaning/purpose.	☐	☐	☐	☐	☐

Do you engage in volunteering activity?

- ○ Yes
- ○ No

Is this since the pandemic?

- ○ Yes
- ○ No

Please select the response that best represents your personal experience at the moment.

Do you have the feeling that you are in an unfamiliar situation and don’t know what to do?

Do you have mixed-up feelings and ideas?

How often do you have feelings that you’re not sure you can keep under control?

Does it happen that you have feelings inside you would rather not feel?

Do you have the feeling that you’re being treated unfairly?

Do you have the feeling that you don’t really care about what goes on around you?

On a scale from 1 (you saw things in the right proportion) to 5 (you overestimated or

underestimated its importance), please select the response that best represents your personal experience.

When something happened, have you generally found that:

On a scale from 1 (you have very clear goals and purpose) to 5 (you have no goals or purpose), please select the response that best represents your personal experience.

Until now your life has had:

On a scale from 1 (a source of deep pleasure and satisfaction) to 5 (a source of pain and boredom), please select the response that best represents your personal experience.

Doing the things you do every day is:

Has it happened in the past that you were surprised by the behaviour of people whom you thought you knew well?

Has it happened that people whom you counted on disappointed you?

How often do you have the feeling that there’s little meaning in the things you do in your daily life?

Many people sometimes feel like losers in certain situations. How often have you felt this way in the past?

Rate the following statements in relation to your personal experience at the moment.

	Strongly agree	Agree	Mixed or neither agree nor disagree	Disagree	Strongly disagree
I feel changes at work generally have positive implications.	☐	☐	☐	☐	☐
I feel that I am generally accepting of changes at work.	☐	☐	☐	☐	☐
I would consider myself open to changes at work.	☐	☐	☐	☐	☐
I can handle job and organisational changes effectively.	☐	☐	☐	☐	☐

I am able to adapt to changing circumstances at work.	☐	☐	☐	☐	☐

If you found any of the questions distressing, we suggest that you contact support services such as Samaritans (116123), Mind (www.mind.org.uk 0300 123 3393), ACAS (www.acas.org.uk 0300 123 1100), Anxiety UK (www.anxietyuk.org.uk 03444775774).

About you

This section will ask you a series of questions about your feelings about life and personal activities.

To what degree have you experienced changes in your feelings as a result of COVID-19?

	A great degree	A moderate degree	Some degree	A small degree	Not at all
A feeling that my future success depends on me.	☐	☐	☐	☐	☐
A sense of optimism.	☐	☐	☐	☐	☐
A feeling that I have control over my life.	☐	☐	☐	☐	☐
A feeling that my life has meaning/purpose.	☐	☐	☐	☐	☐

Do you engage in volunteering activity?

- ○ Yes
- ○ No

Is this since the pandemic?

- ○ Yes
- ○ No

Please select the response that best represents your personal experience at the moment.

Do you have the feeling that you are in an unfamiliar situation and don’t know what to do?

Do you have mixed-up feelings and ideas?

How often do you have feelings that you’re not sure you can keep under control?

Does it happen that you have feelings inside you would rather not feel?

Do you have the feeling that you’re being treated unfairly?

Do you have the feeling that you don’t really care about what goes on around you?

On a scale from 1 (you saw things in the right proportion) to 5 (you overestimated or

underestimated its importance), please select the response that best represents your personal experience.

When something happened, have you generally found that:

On a scale from 1 (you have very clear goals and purpose) to 5 (you have no goals or purpose), please select the response that best represents your personal experience.

Until now your life has had:

On a scale from 1 (a source of deep pleasure and satisfaction) to 5 (a source of pain and boredom), please select the response that best represents your personal experience.

Doing the things you do every day is:

Has it happened in the past that you were surprised by the behaviour of people whom you thought you knew well?

Has it happened that people whom you counted on disappointed you?

How often do you have the feeling that there’s little meaning in the things you do in your daily life?

Many people sometimes feel like losers in certain situations. How often have you felt this way in the past?

If you found any of the questions distressing, we suggest that you contact support services such as Samaritans (116123), Mind (www.mind.org.uk 0300 123 3393), ACAS (www.acas.org.uk 0300 123 1100), Anxiety UK (www.anxietyuk.org.uk 03444775774).

Final thoughts

Further Details

While you were providing your responses to the questions, were there any thoughts that you would like to share with us, or do you have anything further to add?

If you would also like to be contacted by one of the research team about a further interview to discuss your experiences in relation to work/employment since the COVID-19 pandemic please tick this box:

- Yes, I would like to be contacted about a further interview.

If you found any of the questions distressing, we suggest that you contact support services such as Samaritans (116123), Mind (www.mind.org.uk 0300 123 3393), ACAS (www.acas.org.uk 0300 123 1100), Anxiety UK (www.anxietyuk.org.uk 03444775774).

Thank you

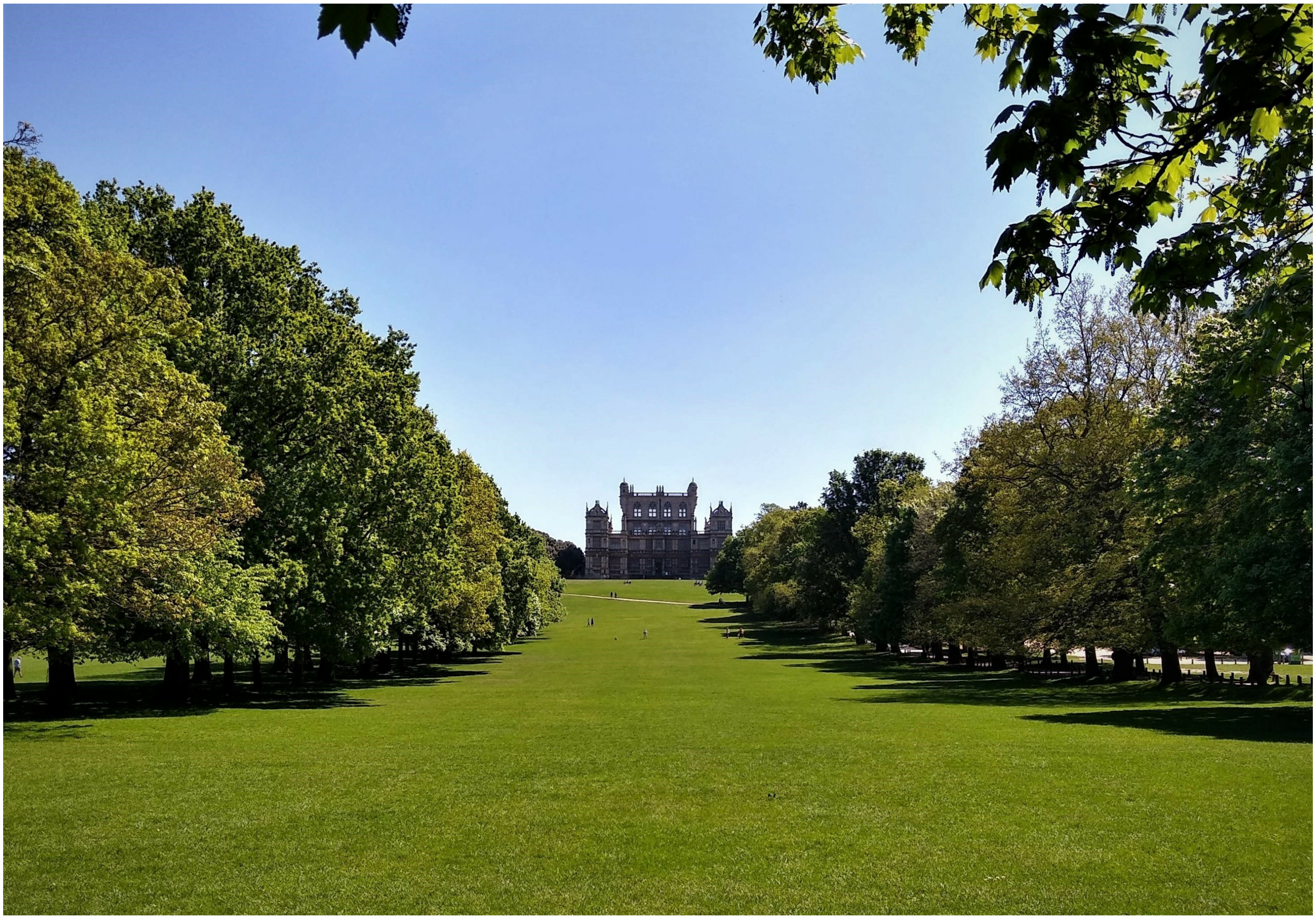

Thank you very much for taking part in this survey for the WoW study. We really do appreciate you taking the time to contribute to this research during these challenging times.

If you have provided us with your email address, we will be back in touch in two weeks with the next survey, which will be shorter than the one you have just completed.

For information about looking after your well-being, visit Every Mind Matters.

Want to know more about the project and hear about our findings?

Visit our WoW Study website
Follow us on Twitter: @WoWStudyUK
Find us on Facebook: fb.me/WoWStudyUK

Best wishes,

The WoW Study Research Team

If you found any of the questions distressing, we suggest that you contact support services such as Samaritans (116123), Mind (www.mind.org.uk 0300 123 3393), ACAS (www.acas.org.uk 0300 123 1100), Anxiety UK (www.anxietyuk.org.uk 03444775774).

Key for selection options

3 - What is your gender?

- Female
- Male
- Non-binary
- Prefer not to say

4 - What is your ethnic group?

- Arab
- Asian
- Black/African/Caribbean
- Mixed/Multiple ethnic groups
- White
- Other ethnic groups

5 - What is your geographical location in the United Kingdom?

- North
- Midlands
- East
- London
- South
- Wales
- Scotland
- Northern Ireland

6 - What is your relationship status?

Single
Married
In a civil partnership
Divorced or separated
Widowed
Prefer not to say

7 - Are you self-isolating:

By yourself
With family
With friends
Other

8 - Are you self-isolating in a:

House
Flat
Shared home or flat
Other

11.a.ii - How many dependents do you have living with you?

None
1
2
3
4
5
more than five
prefer not to say
not applicable

13 - What is the nature of your employment contract?

Full-time permanent
Part-time permanent
Full-time fixed term
Part-time fixed term
Zero-hours contract
Other

14 - Please select the option that best represents what you do in your main paid job?

By main job, we mean the one you spend most of your working hours.

- Managers, Directors and Senior Officials
- Professional Occupations
- Associate Professional and Technical Occupations
- Administrative and Secretarial Occupations
- Skilled Trades Occupations
- Caring, Leisure and Other Service Occupations
- Sales and Customer Service Occupations
- Process, Plant and Machine Operatives
- Elementary Occupations
- Other

15 - Please select the option that best describes the industry your organisation is in.

- Accommodation and food services
- Agriculture, forestry and fishing
- Arts, entertainment, recreation and other services
- Business administration and support services
- Construction
- Education
- Finance and insurance
- Health
- Information and communication
- Manufacturing
- Mining, quarrying and utilities
- Motor trades
- Motor vehicle (wholesale, retail, repair)
- Production
- Professional, scientific and technical
- Property
- Public administration and defence
- Retail
- Transport, storage, postal
- Wholesale
- Other

16 - Are you working in the…?

- Private sector
- Public sector
- Joint public-private sector or company
- Not for profit sector, NGO
- Other

17 - How would you describe the size of your organisation?

- 1 (you work alone)
- Very small (between 2 to 10 people)
- Small (less than 50 people)
- Medium (51 - 250)
- Large (more than 250)

18 - Are you a key worker?

- Yes
- No
- Prefer not to say

19 - Do you manage or supervise anyone at work?

- Yes
- No
- Prefer not to say

21 - What is the nature of your employment contract?

- Full-time permanent
- Part-time permanent
- Full-time fixed term
- Part-time fixed term
- Other

22 - Please select the option that best represents what you do in your main paid job? By main job, we mean the one you spend most of your working hours.

- Managers, Directors and Senior Officials
- Professional Occupations
- Associate Professional and Technical Occupations
- Administrative and Secretarial Occupations
- Skilled Trades Occupations
- Caring, Leisure and Other Service Occupations
- Sales and Customer Service Occupations
- Process, Plant and Machine Operatives
- Elementary Occupations
- Other

23 - Please select the option that best describes the industry your organisation is in.

- Accommodation and food services

Agriculture, forestry and fishing
Arts, entertainment, recreation and other services
Business administration and support services
Construction
Education
Finance and insurance
Health
Information and communication
Manufacturing
Mining, quarrying and utilities
Motor trades
Motor vehicle (wholesale, retail, repair)
Production
Professional, scientific and technical
Property
Public administration and defence
Retail
Transport, storage, postal
Wholesale
Other

24 - Are you working in the…?

Private sector
Public sector
Joint public-private sector or company
Not for profit sector, NGO
Other

25 - How would you describe the size of your organisation?

1 (you work alone)
Very small (between 2 to 10 people)
Small (less than 50 people)
Medium (51 - 250)
Large (more than 250)

26 - Are you a key worker?

Yes
No
Prefer not to say

27 - Do you manage or supervise anyone at work?

Yes
No
Prefer not to say

32 - In general, how satisfied are you with your life?

Very satisfied
Satisfied
Neither satisfied nor dissatisfied
Dissatisfied
Very dissatisfied

33 - How is your health in general? Would you say it is…

Very good
Good
Fair
Bad
Very bad
No opinion
Prefer not to say

37 - In general, how satisfied are you with your life?

Very satisfied
Satisfied
Neither satisfied nor dissatisfied
Dissatisfied
Very dissatisfied

38 - How is your health in general? Would you say it is…

Very good
Good
Fair
Bad
Very bad
No opinion
Prefer not to say

41 - In general, how satisfied are you with your life?

Very satisfied

Satisfied
Neither satisfied nor dissatisfied
Dissatisfied
Very dissatisfied

42 - How is your health in general? Would you say it is…
Very good
Good
Fair
Bad
Very bad
No opinion
Prefer not to say

46 - In general, how satisfied are you with your life?
Very satisfied
Satisfied
Neither satisfied nor dissatisfied
Dissatisfied
Very dissatisfied

47 - How is your health in general? Would you say it is…
Very good
Good
Fair
Bad
Very bad
No opinion
Prefer not to say

55 - How often have you considered leaving your job?
Never
Rarely
Sometimes
Very often
Always

56 - To what extent is your current job satisfying your personal needs?
To a very large extent

To a large extent
To a moderate extent
To some extent
To no extent

57 - How often are you frustrated when not given the opportunity at work to achieve your personal work-related goals?

Never
Rarely
Sometimes
Very often
Always

58 - How often do you dream about getting another job that will better suit your personal needs?

Never
Rarely
Sometimes
Very often
Always

59 - How likely are you to accept another job at the same level of pay should it be offered to you?

Highly unlikely
Unlikely
Somewhat likely or unlikely
Likely
Very likely

60 - How often do you look forward to another day at work?

Never
Rarely
Sometimes
Very often
Always

66 - How often have you considered leaving your job?

Never
Rarely

Sometimes
Very often
Always

67 - To what extent is your current job satisfying your personal needs?
To a very large extent
To a large extent
To a moderate extent
To some extent
To no extent

68 - How often are you frustrated when not given the opportunity at work to achieve your personal work-related goals?
Never
Rarely
Sometimes
Very often
Always

69 - How often do you dream about getting another job that will better suit your personal needs?
Never
Rarely
Sometimes
Very often
Always

70 - How likely are you to accept another job at the same level of pay should it be offered to you?
Highly unlikely
Unlikely
Somewhat likely or unlikely
Likely
Very likely

71 - How often do you look forward to another day at work?
Never
Rarely
Sometimes

Very often
Always

75 - How financially secure do you feel at the moment?
Very secure
Secure
Somewhat secure
Not secure
Not at all secure

79 - How financially secure do you feel at the moment?
Very secure
Secure
Somewhat secure
Not secure
Not at all secure

84 - Do you have the feeling that you are in an unfamiliar situation and don’t know what to do?
Very seldom or never
Seldom
Sometimes
Often
Very often

85 - Do you have mixed-up feelings and ideas?
Very seldom or never
Seldom
Sometimes
Often
Very often

86 - How often do you have feelings that you’re not sure you can keep under control?
Very seldom or never
Seldom
Sometimes
Often
Very often

87 - Does it happen that you have feelings inside you would rather not feel?

Very seldom or never
Seldom
Sometimes
Often
Very often

88 - Do you have the feeling that you're being treated unfairly?

Very seldom or never
Seldom
Sometimes
Often
Very often

89 - Do you have the feeling that you don't really care about what goes on around you?

Very seldom or never
Seldom
Sometimes
Often
Very often

90 - When something happened, have you generally found that:

1 - You saw things in the right proportion
2
3
4
5 - You overestimated or underestimated its importance

91 - Until now your life has had:

1 - Very clear goals and purpose
2
3
4
5 - No goals or purpose

92 - Doing the things you do every day is:

1- A source of deep pleasure and satisfaction
2
3

4
5 - A source of pain and boredom

93 - Has it happened in the past that you were surprised by the behaviour of people whom you thought you knew well?

Never happened
Rarely
Sometimes
Very often
Always happened

94 - Has it happened that people whom you counted on disappointed you?

Never happened
Rarely
Sometimes
Very often
Always happened

95 - How often do you have the feeling that there's little meaning in the things you do in your daily life?

Very seldom or never
Seldom
Sometimes
Often
Very often

96 - Many people sometimes feel like losers in certain situations. How often have you felt this way in the past?

Never
Seldom
Sometimes
Often
Very often

100 - Do you have the feeling that you are in an unfamiliar situation and don't know what to do?

Very seldom or never
Seldom
Sometimes

Often
Very often

101 - Do you have mixed-up feelings and ideas?
Very seldom or never
Seldom
Sometimes
Often
Very often

102 - How often do you have feelings that you’re not sure you can keep under control?
Very seldom or never
Seldom
Sometimes
Often
Very often

103 - Does it happen that you have feelings inside you would rather not feel?
Very seldom or never
Seldom
Sometimes
Often
Very often

104 - Do you have the feeling that you’re being treated unfairly?
Very seldom or never
Seldom
Sometimes
Often
Very often

105 - Do you have the feeling that you don’t really care about what goes on around you?
Very seldom or never
Seldom
Sometimes
Often
Very often

106 - When something happened, have you generally found that:

1 - You saw things in the right proportion
2
3
4
5 - You overestimated or underestimated its importance

107 - Until now your life has had:

1 - Very clear goals and purpose
2
3
4
5 - No goals or purpose

108 - Doing the things you do every day is:

1- A source of deep pleasure and satisfaction
2
3
4
5 - A source of pain and boredom

109 - Has it happened in the past that you were surprised by the behaviour of people whom you thought you knew well?

Never happened
Rarely
Sometimes
Very often
Always happened

110 - Has it happened that people whom you counted on disappointed you?

Never happened
Rarely
Sometimes
Very often
Always happened

111 - How often do you have the feeling that there's little meaning in the things you do in your daily life?

Very seldom or never

- Seldom
- Sometimes
- Often
- Very often

112 - Many people sometimes feel like losers in certain situations. How often have you felt this way in the past?

- Never
- Seldom
- Sometimes
- Often
- Very often
