# Supplementary material for: Psychological detachment from work predicts mental wellbeing of working-age adults: Findings from the ‘Wellbeing of the Workforce’ (WoW) prospective longitudinal cohort study
Source: PLoS One. 2025 Jan 14;20(1):e0312673. doi: 10.1371/journal.pone.0312673 (PMC11731735; doi:10.1371/journal.pone.0312673)
Supplement: S2 Text — (PDF) [file pone.0312673.s002.pdf]

The Well-being of the Workforce

Participant Information and Consent

Ethics Reference Number: 03-0420

Thank you for completing the Wellbeing of the Workforce (WOW) during the COVID-19 crisis study last year. The information you provided has helped us to better understand how our experiences at home and work relate to our well-being and health.

We would like to invite you to complete this short follow-up survey. This survey is much shorter than the last, taking less than 10 minutes to complete. To thank you for your participation in this follow up survey you will be given the opportunity to be entered into a prize draw for £250 of gift vouchers.

The information provided through this survey will help us better understand how worker's experiences at home and work have changed over time and their relationship to well-being.

Your participation in this study is entirely voluntary and you can withdraw at any time by clicking the browser exit button. Please note, the data will only be uploaded on completion of the questionnaire by clicking the SUBMIT button. You are free to omit any question.

For more information on why you have been invited to participate in this follow-up survey, what it involves, and contact details of the research team, please read the attached PDF file.

[link to PDF file]

By clicking 'next' you are providing your consent to participate in this follow-up survey.

Your work status

Since June 2020, has your work status changed? If yes, how?

Which of the following most accurately describes your current work status for your main job? Your response to this answer is required as it allows us to tailor the survey to your individual circumstances. ✱ *Required*

- Employed and working
- Made redundant
- Self-employed and still working
- Unemployed
- On a zero-hours contract/temporary contract
- Other
- Employed and furloughed (job retention scheme)
- Employed and flexi-furloughed (flexible furlough scheme)

If you selected Other, please specify:

If you found any of the questions distressing, we suggest that you contact support services such as Samaritans (116123), Mind (www.mind.org.uk 0300 123 3393), ACAS (www.acas.org.uk 0300 123 1100), Anxiety UK (www.anxietyuk.org.uk 03444775774).

Your well-being

In this section, we will ask you a series of questions about your overall well-being.

Please indicate for each of the five statements, which is closest to how you have been feeling over the last two weeks.

	All of the time	Most of the time	More than half of the time	Less than half of the time	Some of the time	At no time
I have felt cheerful and in good spirits.	○	○	○	○	○	○
I have felt calm and relaxed.	○	○	○	○	○	○
I have felt active and vigorous.	○	○	○	○	○	○
I woke up feeling fresh and rested.	○	○	○	○	○	○
My daily life has been filled with things that interest me.	○	○	○	○	○	○

In general, how satisfied are you with your life?

- ○ Very satisfied
- ○ Satisfied
- ○ Neither satisfied nor dissatisfied
- ○ Dissatisfied
- ○ Very dissatisfied

How is your health in general? Would you say it is…

- Very good
- Good
- Fair
- Bad
- Very bad
- No opinion
- Prefer not to say

Over the last two weeks, how often have you been bothered by the following problems?

	Not at all	Several days	More than half the days	Nearly every day
Feeling nervous, anxious or on edge	○	○	○	○
Not being able to stop or control worrying	○	○	○	○
Worrying too much about different things	○	○	○	○
Trouble relaxing	○	○	○	○
Being so restless that it is hard to sit still	○	○	○	○
Becoming easily annoyed or irritable	○	○	○	○
Feeling afraid as if something awful might happen	○	○	○	○

If you found any of the questions distressing, we suggest that you contact support services such as Samaritans (116123), Mind (www.mind.org.uk 0300 123 3393), ACAS (www.acas.org.uk 0300 123 1100), Anxiety UK (www.anxietyuk.org.uk 03444775774).

Your well-being

In this section, we will ask you a series of questions about your overall well-being.

Please indicate for each of the five statements, which is closest to how you have been feeling over the last two weeks.

	All of the time	Most of the time	More than half of the time	Less than half of the time	Some of the time	At no time
I have felt cheerful and in good spirits.	○	○	○	○	○	○
I have felt calm and relaxed.	○	○	○	○	○	○
I have felt active and vigorous.	○	○	○	○	○	○
I woke up feeling fresh and rested.	○	○	○	○	○	○
My daily life has been filled with things that interest me.	○	○	○	○	○	○

In general, how satisfied are you with your life?

- ○ Very satisfied
- ○ Satisfied
- ○ Neither satisfied nor dissatisfied
- ○ Dissatisfied
- ○ Very dissatisfied

How is your health in general? Would you say it is…

- Very good
- Good
- Fair
- Bad
- Very bad
- No opinion
- Prefer not to say

Over the last two weeks, how often have you been bothered by the following problems?

	Not at all	Several days	More than half the days	Nearly every day
Feeling nervous, anxious or on edge	○	○	○	○
Not being able to stop or control worrying	○	○	○	○
Worrying too much about different things	○	○	○	○
Trouble relaxing	○	○	○	○
Being so restless that it is hard to sit still	○	○	○	○
Becoming easily annoyed or irritable	○	○	○	○
Feeling afraid as if something awful might happen	○	○	○	○

If you found any of the questions distressing, we suggest that you contact support services such as Samaritans (116123), Mind (www.mind.org.uk 0300 123 3393), ACAS (www.acas.org.uk 0300 123 1100), Anxiety UK (www.anxietyuk.org.uk 03444775774).

Your well-being

In this section, we will ask you a series of questions about your overall well-being.

Please indicate for each of the five statements, which is closest to how you have been feeling over the last two weeks.

	All of the time	Most of the time	More than half of the time	Less than half of the time	Some of the time	At no time
I have felt cheerful and in good spirits.	○	○	○	○	○	○
I have felt calm and relaxed.	○	○	○	○	○	○
I have felt active and vigorous.	○	○	○	○	○	○
I woke up feeling fresh and rested.	○	○	○	○	○	○
My daily life has been filled with things that interest me.	○	○	○	○	○	○

In general, how satisfied are you with your life?

- ○ Very satisfied
- ○ Satisfied
- ○ Neither satisfied nor dissatisfied
- ○ Dissatisfied
- ○ Very dissatisfied

How is your health in general? Would you say it is…

- Very good
- Good
- Fair
- Bad
- Very bad
- No opinion
- Prefer not to say

Over the last two weeks, how often have you been bothered by the following problems?

	Not at all	Several days	More than half the days	Nearly every day
Feeling nervous, anxious or on edge	○	○	○	○
Not being able to stop or control worrying	○	○	○	○
Worrying too much about different things	○	○	○	○
Trouble relaxing	○	○	○	○
Being so restless that it is hard to sit still	○	○	○	○
Becoming easily annoyed or irritable	○	○	○	○
Feeling afraid as if something awful might happen	○	○	○	○

If you found any of the questions distressing, we suggest that you contact support services such as Samaritans (116123), Mind (www.mind.org.uk 0300 123 3393), ACAS (www.acas.org.uk 0300 123 1100), Anxiety UK (www.anxietyuk.org.uk 03444775774).

Detachment from work

To what degree do you agree with the following statements. At the end of my working day...

	I strongly agree	I agree	Mixed or neither agree nor disagree	I disagree	I strongly disagree
I forget about work.	○	○	○	○	○
I do not think about work at all.	○	○	○	○	○
I distance myself from my work.	○	○	○	○	○
I get a break from the demands of work.	○	○	○	○	○

If you found any of the questions distressing, we suggest that you contact support services such as Samaritans (116123), Mind (www.mind.org.uk 0300 123 3393), ACAS (www.acas.org.uk 0300 123 1100), Anxiety UK (www.anxietyuk.org.uk 03444775774).

Detachment from work

To what degree do you agree with the following statements. At the end of my working day...

	I strongly agree	I agree	Mixed or neither agree nor disagree	I disagree	I strongly disagree
I forget about work.	○	○	○	○	○
I do not think about work at all.	○	○	○	○	○
I distance myself from my work.	○	○	○	○	○
I get a break from the demands of work.	○	○	○	○	○

If you found any of the questions distressing, we suggest that you contact support services such as Samaritans (116123), Mind (www.mind.org.uk 0300 123 3393), ACAS (www.acas.org.uk 0300 123 1100), Anxiety UK (www.anxietyuk.org.uk 03444775774).

Your work and workplace 1

Please select the response that best represents your experiences at work and in your workplace.

My work has changed in the following ways:

	Increased a lot	Increased slightly	Same as usual	Decreased slightly	Decreased a lot
The number of hours you work per week.	◯	◯	◯	◯	◯
Your salary or income.	◯	◯	◯	◯	◯
The amount of influence you have over your work.	◯	◯	◯	◯	◯
Your task and duties.	◯	◯	◯	◯	◯

To what degree do you agree with the following statements? If not applicable, please leave blank.

	Always	Often	Sometimes	Seldom	Never/ hardly ever
Is your workload unevenly distributed so it piles up?	◯	◯	◯	◯	◯
How often do you not have time to complete all your work tasks?	◯	◯	◯	◯	◯
Do you get behind with your work?	◯	◯	◯	◯	◯
Do you have enough time for your work tasks?	◯	◯	◯	◯	◯
Do you have a large degree of influence on the decisions concerning your work?	◯	◯	◯	◯	◯

Can you influence the amount of work assigned to you?	○	○	○	○	○
Do you have any influence on what you do at work?	○	○	○	○	○
Do you have any influence on how you do your work?	○	○	○	○	○
Are you worried about becoming unemployed?	○	○	○	○	○
Are you worried about being made redundant?	○	○	○	○	○
Are you worried about it being difficult for you to find another job if you became unemployed?	○	○	○	○	○

To what degree do you agree with the following statement.

	Always	Often	Sometimes	Seldom	Never/ hardly ever
My organisation is more family-friendly than most other organisations I could work for.	○	○	○	○	○

To what extent do you agree with the following questions?

	To a very large extent	To a large extent	Somewhat	To a small extent	To a very small extent
Do you enjoy telling others about your place of work?	○	○	○	○	○
Do you feel that your place of work is of great importance to you?	○	○	○	○	○

Would you recommend other people to apply for a position at your workplace?	○	○	○	○	○

If you found any of the questions distressing, we suggest that you contact support services such as Samaritans (116123), Mind (www.mind.org.uk 0300 123 3393), ACAS (www.acas.org.uk 0300 123 1100), Anxiety UK (www.anxietyuk.org.uk 03444775774).

Your work and workplace

Please select the response that best represents your experience.

My work has changed in the following ways:

	Increased a lot	Increased slightly	Same as usual	Decreased slightly	Decreased a lot
The number of hours you work per week.	○	○	○	○	○
Your salary or income.	○	○	○	○	○
The amount of influence you have over your work.	○	○	○	○	○
Your task and duties.	○	○	○	○	○

If you found any of the questions distressing, we suggest that you contact support services such as Samaritans (116123), Mind (www.mind.org.uk 0300 123 3393), ACAS (www.acas.org.uk 0300 123 1100), Anxiety UK (www.anxietyuk.org.uk 03444775774).

Your work and workplace 2

Please read each question and select the response that best represents your personal experience.

How often have you considered leaving your job?

- Never
- Rarely
- Sometimes
- Very often
- Always

To what extent is your current job satisfying your personal needs?

- To a very large extent
- To a large extent
- To a moderate extent
- To some extent
- To no extent

How often are you frustrated when not given the opportunity at work to achieve your personal work-related goals?

- Never
- Rarely
- Sometimes
- Very often
- Always

How often do you dream about getting another job that will better suit your personal needs?

- Never
- Rarely
- Sometimes
- Very often
- Always

How likely are you to accept another job at the same level of pay should it be offered to you?

- Highly unlikely
- Unlikely
- Somewhat likely or unlikely
- Likely
- Very likely

How often do you look forward to another day at work?

- Never
- Rarely
- Sometimes
- Very often
- Always

If you found any of the questions distressing, we suggest that you contact support services such as Samaritans (116123), Mind (www.mind.org.uk 0300 123 3393), ACAS (www.acas.org.uk 0300 123 1100), Anxiety UK (www.anxietyuk.org.uk 03444775774).

Home and work life

The next five questions concern the ways in which your work affects your private life. To what extent do you agree with the following statements:

	To a very large extent	To a large extent	To a moderate extent	To a small extent	To a very small extent
Are there times when you need to be attending to work tasks and home tasks at the same time?	○	○	○	○	○
Do you feel that your work drains so much of your energy that it has a negative effect on your private life?	○	○	○	○	○
Do you feel that your work takes so much of your time that it has a negative effect on your private life?	○	○	○	○	○
The demands of my work interfere with my private and family life.	○	○	○	○	○
Due to work-related duties, I have to make changes to my plans for private and family activities.	○	○	○	○	○

If you found any of the questions distressing, we suggest that you contact support services such as Samaritans (116123), Mind (www.mind.org.uk 0300 123 3393), ACAS (www.acas.org.uk 0300 123 1100), Anxiety UK (www.anxietyuk.org.uk 03444775774).

Experiences at home and about you

To what extent do you agree with the following questions.

	Always	Very Often	Sometimes	Rarely	Never
Do you find that you are busy at home?	○	○	○	○	○
Do you have to do many things in a hurry when you are at home?	○	○	○	○	○
Do you have to carry out a lot of tasks at home (household/caring tasks)?	○	○	○	○	○
How often do issues arise at home that are emotionally demanding?	○	○	○	○	○
How often do your demands at home confront you with things that touch you personally?	○	○	○	○	○
How often do you get frustrated about things concerning your home-life?	○	○	○	○	○
Do you find that you have to plan and organise a lot of things in relation to your home life?	○	○	○	○	○
Do you have to remember a lot of things with regard to your home life?	○	○	○	○	○
Do you have to do many things simultaneously at home?	○	○	○	○	○
Do you have to coordinate everything carefully at home?	○	○	○	○	○

How financially secure do you feel?

- ○ Very secure
- ○ Secure
- ○ Somewhat secure
- ○ Not secure

Not at all secure

To what degree do you agree with the following statements.

	A great degree	A moderate degree	Some degree	A small degree	Not at all
A feeling that my future success depends on me.					
A sense of optimism.					
A feeling that I have control over my life.					
A feeling that my life has meaning/purpose.					

If you found any of the questions distressing, we suggest that you contact support services such as Samaritans (116123), Mind (www.mind.org.uk 0300 123 3393), ACAS (www.acas.org.uk 0300 123 1100), Anxiety UK (www.anxietyuk.org.uk 03444775774).

Final thoughts

While you were providing your responses to the questions, were there any thoughts that you would like to share with us, or do you have anything further to add?

Thank you

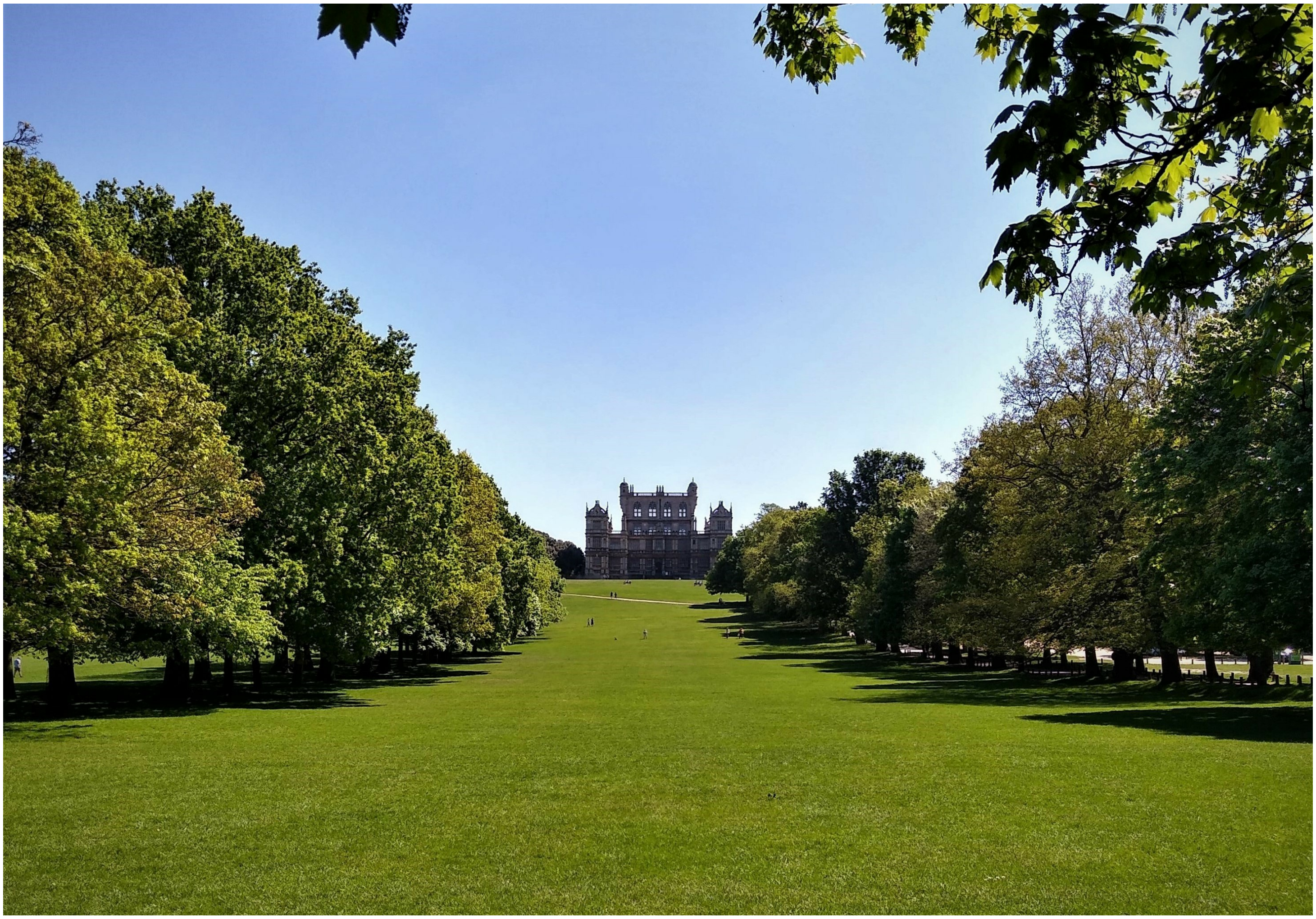

Thank you very much for taking part in this follow-up survey for the WoW study. We really do appreciate you taking the time to continue to contribute to this research during these challenging times.

We will be back in touch in the spring with the last survey, which will be shorter than the one you have just completed.

For information about looking after your well-being, visit Every Mind Matters.

Want to know more about the project and hear about our findings?

Visit our WoW Study website
Follow us on Twitter: @WoWStudyUK
Find us on Facebook: fb.me/WoWStudyUK

Best wishes,

The WoW Study Research Team

WoW Well-being of the Workforce
