# Supplementary material for: Psychological detachment from work predicts mental wellbeing of working-age adults: Findings from the ‘Wellbeing of the Workforce’ (WoW) prospective longitudinal cohort study
Source: PLoS One. 2025 Jan 14;20(1):e0312673. doi: 10.1371/journal.pone.0312673 (PMC11731735; doi:10.1371/journal.pone.0312673)
Supplement: S2 Table — (DOCX) [file pone.0312673.s004.docx]

**S2 Table.** Study variables and measures used over time series data collection.

| Construct | Measure | Questionnaire Properties | Example Item | Psychometric properties | Study reliability in WoW sample^a^ | Timepoint^b^ | Work status |
| --- | --- | --- | --- | --- | --- | --- | --- |
| Demographics | | | | | | | |
| Age | Single item | Open-ended | N/A | N/A | N/A | T1 | All |
| Gender | Single item | Open-ended | N/A | N/A | N/A | T1 | All |
| Ethnicity | Single item | White, Black, Asian, Mixed, Other. | N/A | N/A | N/A | T1 | All |
| Geographical Location | Single item | Open-ended | N/A | N/A | N/A | T1 | All |
| Relationship Status | Single item | Open-ended | N/A | N/A | N/A | T1 | All |
| Number of people in household | Single item | Open-ended | N/A | N/A | N/A | T1 | All |
| Caring responsibilities | Single item | Yes/no/prefer not to say | N/A | N/A | N/A | T1 | All |
| Number of dependents | Single item | Open-ended | N/A | N/A | N/A | T1 | All |
| Employment & Volunteering Activity | | | | | | | |
| Work status | Single item | Employed & still working/employed & furloughed/employed & flexi-furloughed/self-employed & still working/ self-employed & not working/made redundant due to COVID-19/zero-hours contract/other | N/A | N/A | N/A | T1 and T2 | All |
| Nature of contract | Single item | Full-time permanent/ part-time permanent/ full-time fixed term/part-time fixed term/zero-hours contract/other | N/A | N/A | N/A | T1 | All |
| Contracted working hours per week | Single item | Open-ended | N/A | N/A | N/A | T1 | All |
| Main job | Single item | Open-ended | N/A | N/A | N/A | T1 | All |
| Industry | Single item | Open-ended | N/A | N/A | N/A | T1 | All |
| Sector | Single item | Private sector/public sector/joint public-private sector/non-profit | N/A | N/A | N/A | T1 | All |
| Size of organsiation | Single item | You work alone/ between 2 and 10 people/ less than 50 people/ 51 – 250/ more than 250 | N/A | N/A | N/A | T1 | All |
| Key worker | Single item | Yes/no/prefer not to say | N/A | N/A | N/A | T1 | All |
| Managerial or supervisory duties | Single item | Yes/no/prefer not to say | N/A | N/A | N/A | T1 | All |
| Years in organisation | Single item | Open-ended | N/A | N/A | N/A | T1 | All |
| Volunteer activity | Single item | Yes/no | N/A | N/A | N/A | T1 | All |
| Measures used in the current study | | | | | | | |
| Psychological Well-being | The WHO 5 Well-being Index | 5 items measured on a scale from 0 “All of the time” to 5 “At no time” | “I have felt cheerful and in good spirits”. | (Topp, Østergaard, Søndergaard, & Bech, 2015). | α= 0.855 | T1 and T2 | All |
| Life Satisfaction | Life Satisfaction Scale | 1 item on a scale from 0 “very satisfied” to 4 “very dissatisfied” | “In general, how satisfied are you with your life?” | (Cheung & Lucas, 2014). | N/A | T1 and T2 | All |
| Anxiety | Generalised Anxiety Disorder Questionnaire (GAD-7) | 7 items measured on a scale from 0 “not at all” to 3 “nearly every day” | “I have felt calm and relaxed” | (Spitzer, Kroenke, Williams, & Löwe, 2006). | α= 0.884 | T1 and T2 | All |
| Psychological Detachment | Recovery Experience Questionnaire | 4 items measured on a scale from 0 “strongly agree” to 4 “strongly disagree” | “I don’t think about work at all” | (Sonnentag & Fritz, 2007). | α= 0.866 | T1 and T2 | Working, self-employed and working |
| General Health | Single item | 1 item measured on a scale from 1 “very bad” to 5 “very good” | “How is your health in general? Would you say it is…” | N/A | N/A | T1 and T2 | All |
| Measures collected in the WoW study | | | | | | | |
| Financial Security | Single item | 1 item on a scale from 0 “Not at all secure” to 4 “Very secure” | “How financially secure do you feel at the moment?” | N/A | N/A | T1 and T2 | All |
| Changes to work | The Copenhagen Psychosocial Questionnaire | 4 single items measured on a scale from 0 “increased a lot” to 5 “decreased a lot” | “The number of work you work per week” | (Pejtersen, Sønderga, Kristensen, Borg, & Bjorner, 2010)^1^ | α = .600  α = .633 | T1 and T2 | Working, self-employed and working |
| Job Demands |  | 3 items measured on a scale from 0 “Never/ Hardly Ever” to 4 “Always”. | “Do you get behind with your work?” |  | α= 0.820 | T1 and T2 | Working |
| Influence at Work |  | 4 items measured on a scale from 0 “Never/ Hardly Ever” to 4 “Always”. | “Do you have an influence on what you do at work?” |  | α= 0.859 | T1 and T2 | Working |
| Job insecurity |  | 3 items. All items measured on a scale from 0 “Never/ Hardly Ever” to 4 “Always”. | “Are you worried about being made redundant?” |  | α= 0.901 | T1 and T2 | Working, furloughed |
| Commitment to the Workplace |  | 3 items measured on a scale from 0 “to a very small extent” to 4 “to a very large extent” | “Do you feel that your place of work is of great importance to you?” |  | α= 0.845 | T1 and T2 | Working, furloughed |
| Work-life conflict |  | 2 items measured on a scale from 0 “to a very small extent” to 4 “to a very large extent” | “Are there times when you need to be attending to work tasks and home tasks at the same time?” |  | α= 0.903 | T1 and T2 | Working and self-employed and working |
| Facilitation of acceptance | Facilitation of Change Scale | 9 items measured on a scale from 0 “Strongly Disagree” to 4 “Strongly Agree” | “Do you feel that you completely understand the reasons that brough about being furloughed?” | (Gagne et al., 2000)^2^ | α= 0.916 | T1 | Furloughed |
| Organisational Family Support | Perceived Organisational Family Support Scale (POFS) | 10-item questionnaire measured on a seven-point Likert scale ranging from 1 “Strongly Disagree” to 7 “Strongly Agree” | “My organisation is understanding when an employee has a conflict between work and family.” | (Jahn, Thompson, & Kopelman, 2003)^3^ | α= 0.950 | T1 and T2 | Working and furloughed |
| Close Person Support | Close person questionnaire | 10-item questionnaire measured on a scale ranging from 0 “Never” to 4 “Very often”. | “How often did you want more practical assistance from him/her?” | (Stansfeld & Marmot, 1992)^4^ | α= 0.568  α= 0.788 | T1 | All |
| Turnover Intentions | Turnover Intention Scale (TIS) | 6 items measured on a scale from 0 “Never” to 4 “Always” | “How often have you considered leaving your job?” | (Bothma & Roodt, 2013)^5^ | α= 0.612 | T1 and T2 | All |
| Personal Resources | Personal resources scale | 4 items, measured on a scale from 0 “not at all” to 4 “to a great degree” | “ I have a feeling that my future success depends on me” | (Baranik et al. 2019)^6^ | α= 0.924 | T1 and T2 | All |
| Sense of coherence | Sense of coherence questionnaire | 13 items ranging from 0 to 4 with lower scores indicating a better sense of coherence. | “Do you have mixed-up feelings and ideas?” | (Antonovsky, 1993)^7^ | α= 0.857 | T1 | All |
| Openness to change | Openness to change questionnaire | 5 items ranging from 0 “Strongly disagree” to 4 “Strongly agree” | “I am able to adapt to changing circumstances at work” | (Fugate & Kinicki, 2008)^8^ | α= 0.863 | T1 | Furloughed |
| Home Demands | Home Demands Scale | 10 item ranging from 0 “Never” to 4 “Always” | “Do you find you are busy at home” | (Peeters et al., 2005)^9^ | α= 0.918 | T1 and T2 | All |

1 Pejtersen JH, Kristensen TS, Borg V, Bjorner JB. The second version of the Copenhagen Psychosocial Questionnaire. Scandinavian journal of public health. 2010 Feb;38(3_suppl):8-24.

2 Gagne M, Koestner R, Zuckerman M. Facilitating acceptance of organizational change: The importance of self‐determination 1. Journal of applied social psychology. 2000 Sep;30(9):1843-52.

3 Jahn EW, Thompson CA, Kopelman RE. Rationale and construct validity evidence for a measure of perceived organizational family support (POFS): Because purported practices may not reflect reality. Community, Work & Family. 2003 Aug 1;6(2):123-40.

4 Stansfeld S, Marmot M. Deriving a survey measure of social support: the reliability and validity of the Close Persons Questionnaire. Social science & medicine. 1992 Oct 1;35(8):1027-35.

5 Bothma CF, Roodt G. The validation of the turnover intention scale. SA journal of human resource management. 2013 Jan 1;11(1):1-2.

6 Baranik LE, Cheung JH, Sinclair RR, Lance CE. What happens when employees are furloughed? A resource loss perspective. Journal of Career Development. 2019 Aug;46(4):381-94.

7 Antonovsky A. The structure and properties of the sense of coherence scale. Social science & medicine. 1993 Mar 1;36(6):725-33.

8 Fugate M, Kinicki AJ. A dispositional approach to employability: Development of a measure and test of implications for employee reactions to organizational change. Journal of Occupational and organizational Psychology. 2008 Sep;81(3):503-27.

9 Peeters MC, Montgomery AJ, Bakker AB, Schaufeli WB. Balancing work and home: How job and home demands are related to burnout. International journal of stress management. 2005 Feb;12(1):43.
