# Supplementary material for: Psychological detachment from work predicts mental wellbeing of working-age adults: Findings from the ‘Wellbeing of the Workforce’ (WoW) prospective longitudinal cohort study
Source: PLoS One. 2025 Jan 14;20(1):e0312673. doi: 10.1371/journal.pone.0312673 (PMC11731735; doi:10.1371/journal.pone.0312673)
Supplement: S3 Table — (DOCX) [file pone.0312673.s005.docx]

**S3 Table.** Testing normality of variables

| Variables | Kolmogorov-Smirnov | | Shapiro-Wilk | | Skewness | Kurtosis |
| --- | --- | --- | --- | --- | --- | --- |
|  | Statistic (df) | Sig | Statistic (df) | Sig | Statistic (Std. Error) | Statistic (Std. Error) |
| Depression T1 | .074 (130) | .078 | .970 (130) | .005 | -.063 (.212) | -.964 (.422) |
| Depression T2 | .093 (130) | .008 | .964 (130) | .002 | .086 (.212) | -1.051 (.422) |
| Depression difference | .114 (130) | .000 | .973 (130) | .012 | .089 (.212) | 1.485 (.422) |
| Life Satisfaction T1 | .289 (130) | .000 | .861 (130) | .000 | -.631 (.212) | -.339 (.422) |
| Life Satisfaction T2 | .272 (130) | .000 | .873 (130) | .000 | -.342 (.212) | -.770 (.422) |
| Life Satisfaction difference | .253 (130) | .000 | .894 (130) | .000 | -.075 (.212) | .323 (.422) |
| Psychological detachment T1 | .117 (130) | .000 | .937 (130) | .000 | .715 (.212) | .053 (.422) |
| Psychological Detachment T2 | .098 (130) | .004 | .976 (130) | .019 | .194 (.212) | -.365 (.422) |
| Psychological Detachment difference | .087 (130) | .018 | .984 (130) | .121 | .276 (.212) | .601 (.422) |
| Health T1 | .251 (130) | .000 | .858 (130) | .000 | -.492 (.212) | .139 (.422) |
| Health T2 | .269 (130) | .000 | .871 (130) | .000 | -.403 (.212) | -.037 (.422) |
| Health difference | .296 (130) | .000 | .843 (130) | .000 | -.327 (.212) | -.083 (.422) |
| Anxiety T1 | .157 (130) | .000 | .892 (130) | .000 | .968 (.212) | .034 (.422) |
| Anxiety T2 | .126 (130) | .000 | .929 (130) | .000 | .658 (.212) | -.510 (.422) |
| Anxiety difference | .148 (130) | .000 | .959 (130) | .000 | -.130 (.212) | 1.608 (.422) |
